# Validation Study of the Child and Youth Resilience Measure (CYRM-28) Among Dutch Youth

**DOI:** 10.3389/fpsyt.2022.637760

**Published:** 2022-05-09

**Authors:** Harrie Jonkman, Maaike van Rooijen, Marte Wiersma, Roel van Goor

**Affiliations:** ^1^Verwey-Jonker Institute, Utrecht, Netherlands; ^2^Inholland University of Applied Sciences, Amsterdam, Netherlands

**Keywords:** resilience, Child and Youth Resilience Measure, transition to adulthood, scale validation, at-risk youth

## Abstract

A validation study of a 28-item Child and Youth Resilience Measure (CYRM-28) among a Dutch sample was presented. A sample of 525 adolescents (16–20 years old) from the CYRM-28 in the Netherlands was analyzed. Descriptive statistics, confirmatory factor analysis (CFA), construct validity analysis, and reliability tests were carried out on data collected to identify and present factor structure, construct validity, and reliability. The CFA suggested a three-structure framework with individual, relational, and contextual subscales. Overall, the results were similar to the results found in other international validation studies measuring resilience among teenagers. Integral support of vulnerable youth needs to fit in with the lives and world of these adolescents in their transition to adulthood. Measuring resilience with the CYRM-28 can be used to assist this. Dutch individual and contextual subscales need further research.

## Background

While it may differ among young people, the transition to adulthood is often disharmonious. Physically, intellectually, socio-emotionally, and neurologically, the adolescent period is a time of crucial development (1–3). Young people like to master new tasks and are pre-eminently capable of realizing changes. However, they are also inclined to take risky and impulsive decisions and make choices that focus on direct rewards rather than worrying about long-term consequences. This period is therefore also referred to as a high-risk phase when young people are more inclined to substance use and behavior that deviates from or violates social norms and standards (1). In addition, they are more likely to develop problems in both their psychological and social functioning (2, 4, 5).

Some adolescents are at additional risk of developing problems in different areas of life when they reach adulthood (4, 5). This developmental period of adolescence also provides an opportunity for exploration, skill attainment, and support for some segments of the population. Without this opportunity, adolescents may become even more vulnerable (6). This applies especially to vulnerable young people, where problems often occur in more than one area of life (7, 8). Problem areas may be associated with arranging finances, housing, daytime activities, physical and mental health (including addiction problems), relationships (with parents or peers), police, and justice (9).

Individual factors and contexts often determine youth vulnerability. Experimental, observational, longitudinal, and etiological studies show that risk and protective factors across these levels are related to youth vulnerability (2, 4, 7, 10–12). An accumulation of risks in different contexts increases their vulnerability, whereas an accumulation of protective factors acts as a buffer that reduces the chance of problems arising and increases the chance of psychosocial wellbeing and social participation (2, 13, 14).

Interventions designed to prevent problems by averting accumulating risk factors and promoting protective factors are identified in the literature. It has been advocated that interventions should focus on strengthening or increasing the protective factors (13, 14). The emphasis is placed on existing positive forces and possibilities and avoiding problem-oriented approaches. To this end, the concept of resilience has been an increasing focus of research into protective factors (15).

## Resilience

Masten and Coatsworth defined resilience as “demonstrated competence in the context of significant adaptation or development challenges” (16). Resilience is related to the extent to which a person can deal more or less successfully with all kinds of life's setbacks. Resilience was initially understood as a characteristic of an individual; however, more recently, a socio-ecological approach has emerged in which resilience is understood as a characteristic of a person in direct connection with his or her living environment (17–19). Resilience relates here to the availability of resources available to a person and the ability to use them in the event of adversity. Ungar (20) described resilience as “the context of exposure to significant adversity, resilience is both the capacity of individuals to navigate their way to the psychological, social, cultural, and physical resources that sustain their wellbeing, and their capacity individually and collectively to negotiate for these resources to be provided in culturally meaningful ways” (p. 225).

Resilience focuses on how young people succeed in dealing with adversity and how they give meaning to their lives by actively using elements at the individual, relational, and environmental levels (18, 20). This approach is in line with substantive transformation in the social domain, in which attention is paid to the individual's strength for resources in his or her immediate (social) environment. An ecological approach is also underpinned by the fact that it ties in with widely supported and researched theories, such as the social systems theory (21) or the bioecological development theory (22). More importantly, there is much empirical evidence that strengthening resilience at the ecological level contributes to preventing psychosocial problems and social failure (18, 23, 24).

This study is part of a longitudinal study in which resilience is used as an overall concept for understanding the personal and social development of adolescents in the phase of transition to adulthood. The overall project sets out to contribute to the quality and effectiveness of professional support and services for (especially vulnerable) adolescents and to understand young people in their development, social environment, and need for support and how they participate and contribute to society. Part of the overall project, and the focus of this study, is to examine the psychometric properties of the 28-item Child and Youth Resilience Measure (CYRM-28) in the Dutch context. This study is based on the first wave of data collected in 2019.

The properties of the CYRM have been examined and supported in several countries with samples of at-risk youth. Lieberberg et al. (25) found support for the CYRM-28 as a reliable and valid self-report instrument that measures three components of resilience processes in the lives of complex needs of youth (individual, relational, and contextual) in Canada. Sanders et al. (27) also examined these properties in a sample of at-risk New Zealand youth. They also showed reliable and good construct validity in a four-factor structure (individual, family, and two contextual factors). Govender et al. (26) did a similar study among South African adolescents and also found a three-factor model (individual/social, familial, and community/spiritual).

## Methods

### Participants

Adolescents and young adults who use youth support services and without additional support needs were the focus of this study as well as adolescents who face different levels of adversity. The sample is also socially and demographically heterogeneous because the adolescents come from different levels in society. They were recruited via professionals working in different forms of secondary education, secondary vocational education schools, and youth care and youth welfare organizations. Some of the schools are especially focused on youth who have dropped out of school previously and on pupils with mild intellectual and/or learning disabilities. Participants resided in one of the three cities located in the Netherlands: Amsterdam, Haarlem, and Rotterdam. These cities, with service and education functions for the larger metropolitan area, are located in the west of the Netherlands.

The research was conducted by researchers with the support of the municipalities and several educational and youth organizations in the cities. Local government and youth organizations of the two biggest cities of the Netherlands (Amsterdam and Rotterdam) and a middle-sized city (Haarlem) were involved in this project. Similarly, mentors, school counselors, youth care coordinators, health professionals, social workers, and school psychologists supported the recruitment of participants. A “snowball method” was also used as an additional recruitment strategy, where adolescents asked their peers whether they would participate in the study.

All participants completed an online Dutch questionnaire under the supervision of the research team. Depending on the intellectual capacities of the participants to fill in the questionnaires, the data were collected in a classroom or face-to-face setting. Prior to data collection, all participants were informed about the purpose of the study, research procedures, privacy, and data management. Participants explicitly consented to the use of collected data for research by signing an informed consent form, in line with Dutch legislation on the use of confidential information about the purpose of the study. Ethical improvement guidelines were taken into account.

### Measures

#### Social and Demographical Background

##### Age

Respondents were asked to provide their specific age in years and months. The age of participants was divided into two categories: 16–17 years and 18–20 years. Respondents younger than 16 years were not included in the analysis.

##### Gender

Respondents were asked to specify whether they were male, female, or not stated.

##### Living Arrangements

Participants were asked about their current living situation. Participants were allowed to give multiple answers: “living with their biological parents,” “living with their siblings,” “living in an elaborated household with step-parents,” “living with their adoptive parents or foster parents,” “living alone,” “living with roommates,” “living with their partner and/or children,” or “living with other people” [e.g., grandparent(s)].

##### Length of Time in Household

Participants were asked how long they had lived in their current household. The options were “more than 5 years,” “3–5 years,” “1–2 years,” and “ <1 year.”

##### Mobility

Participants were asked how many times they had moved in the past 5 years: “1–2 times in past 5 years,” “between 3 and 5 times,” or “more than 5 times.”

##### Family

Participants were asked about who was included in their understanding of family: their “biological parents,” “siblings,” “friends,” “partner,” “step-parents,” “children,” “foster parents,” “adoption parents,” and/or “other” (e.g., grandparents and cousins).

##### Ethnicity

Ethnicity was based on migration status, that is, whether they were born in the Netherlands and/or their parents were born in the Netherlands. If respondents were born in the Netherlands and their parents as well, participants were defined as having “No Migration Background.”

##### Education

Education was assessed as to whether participants were currently enrolled in some form of secondary education program (e.g., a secondary vocational education program, in higher professional education, or different levels of secondary education) or not enrolled in any form of education.

##### Adversity

Additional background information was asked related to their personal and social functioning. Adolescents answered the following 11 questions: “I have enough money to buy what I find important in my daily life,” “I can handle money well,” “I can find a part-time job that suits me,” “I like the people I live with,” “I live in the place I want to live,” “I have good friends,” “I feel myself physically healthy,” “I feel good,” “I obey the law,” “I have my alcohol consumption under control,” and “I have my drug use under control.” They could answer as totally disagree, disagree, disagree/agree, agree, or totally agree. The total score was turned into a variable called adversity. If they answered totally agree or agree to six or more questions, they got a yes on adversity.

##### Support

In addition, adolescents were asked whether they received any formal support from a psychiatrist, a psychologist, or an educationalist.

#### Resilience

The CYRM shortened version was used to measure resilience (18). The 28 items of this resilience measure were translated into Dutch by the research team. The accuracy of the translation was checked by a native English speaker who was also fluent in Dutch. The construct validity of the translated items was qualitatively checked in pilot sessions with a group of eight students in a vocational education setting. Moreover, the translation was checked by two professionals who work with youth, in one case, youth with limited intellectual abilities.

The CYRM comprises 28 items and measures three dimensions: (1) individual, (2) relations with the caregiver, and (3) the broader context and community. Questions that focus on the individual dimension emphasized personal competence, for example, “I cooperate with people around me” or “I try to finish what I start.” The relational dimension consisted of questions regarding physical care, for example, “My caregiver(s) watch me closely,” “If I am hungry, there is enough to eat.” The contextual dimension involved questions related to philosophical beliefs. For example, “Spiritual beliefs are a source of strength for me” and “I participate in organized religious activities.” Participants responded to questions with a 5-point Likert scale, from 1 indicating “not at all” to 5 representing “a lot.”

#### Emerging Adulthood

The emerging adulthood and subscales of the “Inventory of the Dimensions of Emerging Adulthood” (IDEA) (27) were used as part of the resilience validation analysis. Participants had to indicate how they felt about questions focusing on this age period. An example is “Is this period of your life a time of finding out who you are?.” Answers ranged from (1) “strongly disagree” to (4) “strongly agree.” The subscales “Identity Exploration” and “Self-focused” both showed sufficient reliability, with Cronbach's alpha being 0.74 and 0.74, respectively. They are combined to a total Emerging adulthood scale (EmAd-scale), which is used in the analyses. Cronbach's alpha of this EmAd-scale is 0.83.

### Data Analysis

Descriptive analyses, properties, and measurement model analyses were conducted using Stata 15.1 (28–31). Confirmatory factor analysis (CFA) was used to investigate different factor structure analyses of the CYRM-28. CFA provides a tool to test and compare different hypotheses pertaining to different models and theories of behavior (31). The measurement models tested here comprised latent variables found in three earlier validation studies on individual, relational, and contextual components. Multiple fit indices were used here [among them CFA, the Tucker–Lewis index (TLI), root-mean-square error of approximation (RMSEA), and the Akaike information criterion (AIC)] to compare models (32, 33). The most restrictive and reliable model (consistent with theoretical assumptions) was chosen with fit indices that are above the cutoff scores. Consistent with the literature, the following cutoff scores were used: RMSEA (<0.08) and CFI and TLI (≥0.90) (32–34).

A secondary analysis to examine the construct validity of the CYRM-28 was performed. This was assessed by regressing the three subscales of the CYRM-28 onto the similar scales of identity and self-focus of Emerging Adulthood. Group differences by age, gender, and migration background of youth were examined using *t*-tests. Analyses were performed on two age groups (younger and older groups), two gender groups (female and male), and migration background (non-migration and migration groups) (32, 35–37). Reliability, using Cronbach's alpha statistics of the CYRM-28-total scores, and the three subscales (individual, relational, and contextual) were also examined.

## Results

### Descriptives

Half of the adolescent sample (*N* = 253; 49.9%) belonged to the younger age group (16–17 years old), and the other half (*N* = 254, 50.1%) belonged to the older (18, 19, and 20 years old). Slightly more girls (*N* = 282; 53.5%) than boys (*N* = 240; 45.7%) participated. Three participants (0.6%) reported that they were neither female nor male. Most adolescents reported not living alone (*N* = 492; 94.1%). More than three-quarters of the young adults (*N* = 412; 78.8%) had lived more than 5 years in their current household. Approximately half of the adolescent (*N* = 255; 48.8%) sample had not moved in the past 5 years. The majority (*N* = 460; 88%) viewed their biological parents as family; 35% had no migration background (*N* = 183) and were neither first- nor second-generation western or non-western. Almost the whole sample (*N* = 505; 96.9%) were currently enrolled in an education program (Table 1).

**Table 1:** Descriptives of the sample (*N* = 525).

**Background variables**	***N***	**%**	**Missings (%)**
Age (16–17 years)	253	49.9	3.43
Gender (women)	282	53.5	0.38
With whom they live (not alone)	492	94.1	0.38
Length of time (>5 years)	412	78.8	0.38
Mobility (not moved last 5 years)	255	48.8	0.38
Family (biological parents seen as such)	460	88.0	0.38
Ethnicity (no migration background)	183	35.0	0.38
Education (following at this moment)	505	96.6	0.76
Adversity	21	4.0	0
Support	324	62.0	0.6

### Confirmatory Factor Analysis

**Figure 1:**
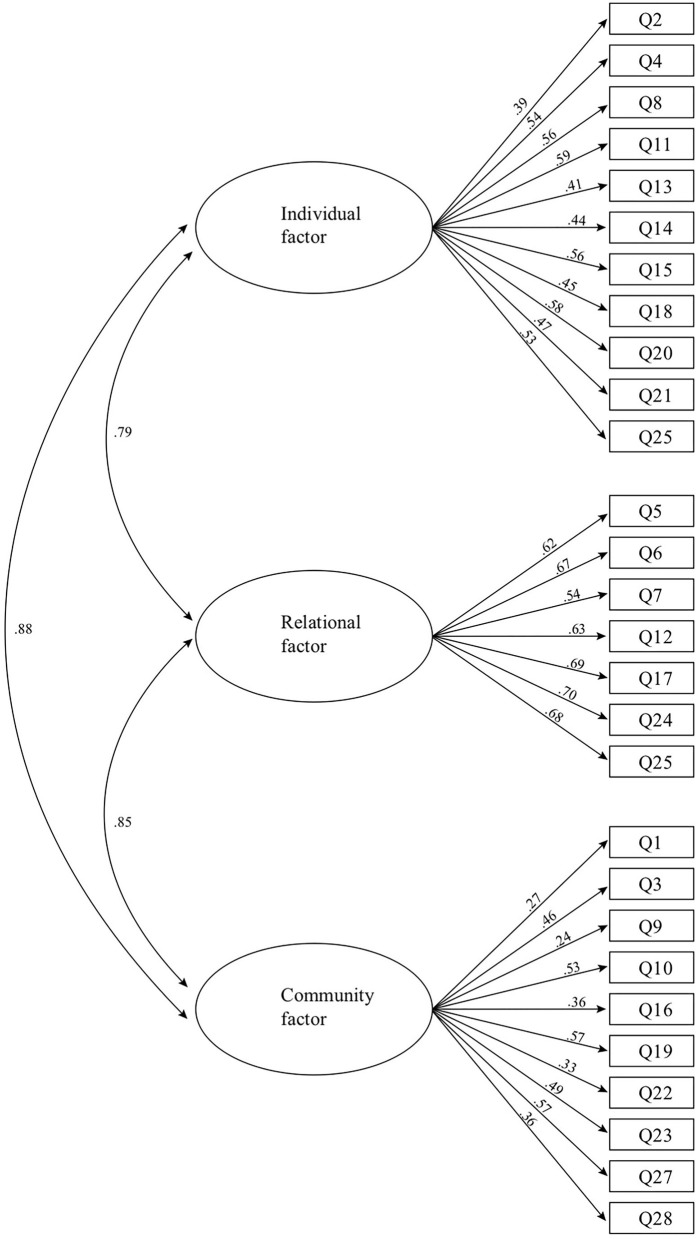
Results of hypothesized model.

In keeping with CFA procedures to improve the overall fit of the model (32, 33), we constrained the error terms of covariance, for instance, between related items like “I feel supported by my friends” and “My friends stand by me during difficult times.” This was done as diagnostic statistics and to examine the correlations between the questions suggested. The questions were highly correlated and thus measured similar constructs (Figure 1). We compared three previously tested models by comparing the fit indices for Model 1 (25), Model 2 (26), and Model 3 (27) to find out which model fitted the data of our sample of Dutch adolescents best. The first model represents the original model based on the theory of Ungar and colleagues. The model of Govender consists of the original three factors but with 24 items instead of 28. The third model consists of 4 factors instead of 3 and includes all 28 items. The results for these models are presented in Table 2.

**Table 2:** Fit indices of the compared models.

	**Chi-square**	**df**	***p*-value**	**CFI**	**TLI**	**RMSEA (90% CFI)**	**AIC**
Model 1: Original 3 factor model (28 items)	939.81	343	<0.001	0.85	0.84	0.058 (−0.54 to 0.063)	37133.012
Model 2: Three factors (24 items)	709.75	247	<0.001	0.87	0.85	0.06 (0.055–0.066)	31546.903
Model 3: Four factors	943.95	340	<0.001	0.85	0.83	0.059 (0.054–0.063)	37143.146

Comparing fit indices of all models, none of the models showed a convincing fit to the data. Although the RMSEA values were moderate, the CFI and TLI remained below their sufficient threshold of 0.90 in all three models. Kenny and McCoach (38) proposed an explanation to these findings as considering the null RMSEA value of each model was below the threshold of 0.158 and the RMSEA value approached 0.05, and it is questionable whether CFI and TLI could produce values above 0.90. Therefore, the interpretation of these fit indices is limited in this study.

Although the AIC of Model 2 suggested a better fit than the other two models, conceptually and in the context of the other fit statistics, the first model was considered to have a better fit overall. Considering the small differences between the three models, we believed Model 1 (Figure 1) was the best fit for the data. This first model also represented the underlying theory of Ungar and colleagues. However, the standardized regression coefficients for the items related to the community scale were relatively low.

### Construct Validity

Regressions indicated that the three CYRM-28 subscales were significantly associated with the identity of the emerging adulthood scale: individual (β = 6.86, *p* < 0.001), relational (β = 15.3, *p* < 0.001), and contextual (β = 11.68, *p* < 0.001). Similarly, the three CYRM-28 subscales were significantly associated with the self-focus scale of Emerging Adulthood: individual (β = 5.74, *p* < 0.001), relational (β = 12.21, *p* < 0.001), and contextual (β = 10.04, *p* < 0.001).

*t*-Tests comparing age and gender groups did not reveal significant differences between groups (Table 3). However, significant differences between migration groups for the contextual subscale and the migration subgroup (*t* = −3.98, *p* < 0.001) were observed.

**Table 3:** *T*-tests on the three subscale scores by age, gender, and migration background.

**Subscales**	**Grouping**	***t-*test scores**	***p*-values**
Individual	Age	0.49	*0.624*
Relational	Age	1.34	*0.179*
Contextual	Age	−1.37	*0.170*
Individual	Gender	0.45	*0.652*
Relational	Gender	−0.22	*0.823*
Contextual	Gender	−1.02	*0.309*
Individual	Migration	0.74	*0.458*
Relational	Migration	−0.54	*0.587*
Contextual	Migration	−3.98	0.000^*^

* *p < 0.05 (significant differences, bold)*.

### Reliability Analyses

Cronbach's alpha for all CYRM-28 items was 0.90 and thus considered strong. There was internal consistency for the individual (α = 0.79), relational (α = 0.84), and contextual/community (α = 0.67) scales. The reliability of the scales, undertaken on one measurement moment, was supported by the results.

## Discussion

This study examined the factor structure, construct validity, and reliability of the CYRM-28 in a group of Dutch adolescents and young adults. Our CFA approach showed a reasonable fit of the model to the data. Our results are consistent with those of Govender et al. (26), who examined the psychometric properties of the CYRM-28 in a sample of South African adolescents. Study findings also suggested that items of the individual and community factor like “Do you have people you look up to?” and “Do you enjoy your community's traditions?” showed low correlation coefficients with the underlying factor.

Other international research studies on adolescents have identified similar results to those for Dutch samples; Dutch youth often define themselves as self-confident and give the community a different meaning compared to adolescents with an Anglo-Saxon background (2). Overall, this study confirmed the suitability of the questionnaire for measuring resilience. The results offered further evidence for the generally accepted resilience measurement instrument CYRM-28.

The study findings suggest that resilience among Dutch adolescents and young adults comprises a three-factor structure: individual, relational, and contextual. It confirms that this three-factor structure resilience framework is a worthwhile structure for measuring it in the Dutch context and can be used in the future. The study also underlines the importance of validating the structure of resilience when translating a developed measurement instrument into another language. This is the first study to have brought evidence on the suitability of the CYRM-28 in a Dutch context. The findings of this study suggest that this instrument can be used in longitudinal studies; it can also be used as part of resilience interventions targeted at vulnerable youth.

Notwithstanding the strengths of the study, the limitations of the study should be noted. The findings concerning the validity of the CYRM-28 in the Dutch context raised some questions that should be investigated in future research, namely, the community subscale. Other limitations include that the sample was restricted to predominantly adolescents from vocational schools in three metropolitan Dutch cities instead of also including rural areas. Moreover, previous studies (26) used data from more than one-time point; this study was cross-sectional.

## Conclusion

Our study has demonstrated that measuring resilience with the CYRM-28 can be used to better understand the living environment, the life course, and perspective of life of, especially vulnerable, young people in the Netherlands. The insights have relevance for policymakers and those who work in the implementation and educational practice.

## Data Availability Statement

The raw data supporting the conclusions of this article will be made available by the authors, without undue reservation.

## Ethics Statement

The studies involving human participants were reviewed and approved by UMC Utrecht WAG/mb/18/0148812. Written informed consent to participate in this study was provided by the participants' legal guardian/next of kin.

## Author Contributions

HJ: study design, supervision, conceptualization, analysis, and writing. MR: study design, conceptualization, analysis, and writing. MW: data collection, data interpretation, and critical review. RG: study design, supervision, data collection, data interpretation, and critical review. All authors approved the final version.

## Funding

The resilience study was funded by a research grant (project number SVB/RAAK.PRO02.080), from SIA/RAAK-PRO (call 2016), and SIA has no role in any part of the research, writing, and reviewing of the manuscript.

## Conflict of Interest

The authors declare that the research was conducted in the absence of any commercial or financial relationships that could be construed as a potential conflict of interest.

## Publisher's Note

All claims expressed in this article are solely those of the authors and do not necessarily represent those of their affiliated organizations, or those of the publisher, the editors and the reviewers. Any product that may be evaluated in this article, or claim that may be made by its manufacturer, is not guaranteed or endorsed by the publisher.

## References

[1] Anderson SL. Commentary on the special issue on the adolescent brain: adolescence, trajectories, and the importance of prevention. Neurosci Biobehav Rev. (2016) 70:329–33. 10.1016/j.neubiorev.2016.07.012 27423540 PMC5268741

[2] Farrington DF Jonkman H Groeger-Roth F. Delinquency and Drug Use. Understanding Risk and Protective Factors. New York, NY: Springer (2021).

[3] van Duijvenvoorde ACK Peters S Braams BR Crone EA. What motivates adolescents? Neural responses to rewards and their influence on adolescents' risk taking, learning, and cognitive control. Neurosci Biobehav Rev. (2016) 70:329–33. 10.1016/j.neubiorev.2016.06.037 27353570

[4] Dryfoos JG. Safe Passage. Making It Through Adolescence in a Risky Society. What Parents, School and Communities Can Do. Oxford: Oxford University Press (1998).

[5] Putnam R. Our Kids. The American Dream in Crisis. New York, NY: Simon & Schuster (2015).

[6] Cosner Berzin S. Vulnerability in the transition to adulthood: definining risk based on youth profiles. Child Youth Serv Rev. (2010) 32:487–95. 10.1016/j.childyouth.2009.11.001

[7] Jonkman H. Some Years of Communities That Care. Learning From a Social Experiment. Amsterdam: VU (2012).

[8] Paulsen V Berg B. Social support and interdependency in transition to adulthood from child welfare services. Child Youth Serv Rev. (2016) 68:125–31. 10.1016/j.childyouth.2016.07.006

[9] Lauriks S Buster M de Wit M van de Weerd S Tigchelaar G Fassaert T. Zelfredzaamheidmatrix. (2013). Available online at: http://www.zelfredzaamheidmatrix.nl/resources/site1449/General/Matrix/Zelfredzaamheid_Matrix_op%20website.pdf (cited October 14, 2016).

[10] Junger Tas J Haen Marshal I Enzmann D Kilias M Steketee M Gruszcynska B. Juvenile Delinquency in Europe and Beyond. Results of the Second International Self-Report Delinquency Study. New York, NY: Springer (2010). 10.1007/978-0-387-95982-5

[11] Loeber R Farrington DF. Serious and Violent Juvenile Offenders: Risk Factors and Successful Interventions. Thousand Oaks, CA: Sage Publications (1998).

[12] Loeber R Slot NW van der Laan P Hoeve M. Tomorrow's Criminals. The Development of Child Delinquency and Effective Interventions. Burlinton, VT: Ashgate (2008).

[13] Catalano RF Berglund ML Ryan JAM Lonczak HS Hawkins JD. Positive youth development in the United States: research findings on evaluations of positive youth development programs. Ann Am Acad Pol Soc Sci. (2004) 31:98–124. 10.1177/0002716203260102

[14] van Yperen T Wijnen B Hageraats R. Evaluatie Jeugdwet. Meer Kwaliteit en Minder Zorgen. Utrecht: Nederlands Jeugdinstituut (2016).

[15] Zolkoski SM Bullock LM. Resilience in children and youth: a review. Child Youth Serv Rev. (2012) 34:2295–303. 10.1016/j.childyouth.2012.08.009

[16] Masten AS Coatsworth JD. The development of competence in favorable and unfavorable environments: lessons from research on successful children. Am Psychol. (1998) 53:205–20. 10.1037/0003-066X.53.2.205 9491748

[17] Ungar M. (editor.). The Social Ecology of Resilience. A Handbook of Theory and Practice. Dordrecht: Springer (2012). 10.1007/978-1-4614-0586-3

[18] Ungar M. The Child and Youth Resilience Measure: Youth Version. User's Manual. Resilience Research Centre (2013). Available online at: http://resilienceresearch.org/ (cited October 25, 2016).

[19] Ungar M. Working With Children and Youth With Complex Needs. 20 Skills to Build Resilience. New York, NY: Routledge (2015). 10.4324/9781315755304

[20] Ungar M. Resilience across cultures. Br J Soc Work. (2008) 38:218–35. 10.1093/bjsw/bcl343

[21] Lewin K. Field Theory in Social Sciences; Selected Theoretical Papers. New York, NY: Harper (1951).

[22] Bronfenbrenner U. Making Human Beings Human: Bioecological Perspectives on Human Development. Thousand Oaks: Sage Publications (2005).

[23] Jaffee SR Caspi A Moffitt TE Polo-Thomas M Taylor A. Individual, family, and neighborhood factors distinguish resilient from non-resilient maltreated children: a cumulative stressors model. Child Abuse Negl. (2007) 31:231–53. 10.1016/j.chiabu.2006.03.011 17395260 PMC1978062

[24] Prilleltensky I. Wellness as fairness. Am J Community Psychol. (2012) 49:1–21. 10.1007/s10464-011-9448-8 21643926

[25] Liebenberg L Ungar M van den Vijver F. Validation of the child and youth resilience measure-28 (CYRM-28) among Canadian youth. Res Soc Work Pract. (2011) 22:219–26. 10.1177/1049731511428619

[26] Govender K Cowden RG Asante KO George G Reardon C. Validation of the child and youth resilience measure among South African adolescents. PLoS ONE. (2017) 12:e0185815. 10.1371/journal.pone.0185815 28982195 PMC5628872

[27] Sanders J Munford R Thimasarn-Anwar T Liebenberg L. Validation of the child and youth resilience measure (CYRM-28) on a sample of at-risk New Zealand youth. Res Soc Work Pract. (2015) 27:827–40. 10.1177/1049731515614102

[28] Acock AC. Structural Equation Modeling Using Stata, Revised Edition. College Station, TX: Stata Corp LP (2013).

[29] Mehmetoglu M Jakobsen TG. Applied Statistics Using Stata. A Guide for the Social Sciences. Los Angeles, CA: Sage (2017).

[30] Mitchell MN. Stata for Behavioural Sciences. College Station, TX: Stata Press (2015).

[31] StataCorp. Stata Statistical Software: Release 15. College Station, TX: StataCorp LP (2017).

[32] Cabrera-Nguyen EP. Author guidelines for reporting scale development and validation results in the journal of the society for social work and research. J Soc Soc Work Res. (2010) 1:99–103. 10.5243/jsswr.2010.8

[33] Kline R. Principles and Practice of Structural Equation Modelling. 3rd ed. New York, NY: Guilford Press (2011).

[34] Schreiber JB Nora A Stage FK Barlow EA King J. Reporting structural equation modeling and confirmatory factor analysis results: a review. J Educ Res. (2006) 99:323–38. 10.3200/JOER.99.6.323-338

[35] Schumacher RE Lomax RG. A Beginner's Guide to Structural Equation Modeling. 4th ed. London: Routledge (2016).

[36] Chou C Bentler P. Estimates and tests in structural equation modeling. In: Hoyle R, editor. Structural Equation Modelling: Concepts, Issues, and Applications. Thousand Oaks, CA: Sage (1995). p. 37–55.

[37] Stevens J. Applied Multivariate Statistics for the Social Sciences. 5th ed. New York, NY: Routledge (2009).

[38] Kenny DA Mccoach DB. Effect of the number of variables on measures of fit in structural equation modeling. Struct Equ Model. (2003) 10:333–51. 10.1207/S15328007SEM1003_1

